# Serum complement proteomics reveal biomarkers for hypertension disorder of pregnancy and the potential role of Clusterin

**DOI:** 10.1186/s12958-021-00742-z

**Published:** 2021-04-19

**Authors:** Shanshui Zeng, Mengru Han, Min Jiang, Fei Liu, Yanwei Hu, Yan Long, Chunyan Zhu, Fangling Zeng, Qiangsheng Gan, Weitao Ye, Wenjin Fu, Hongling Yang

**Affiliations:** 1 Department of Laboratory, Guangzhou Women and Children’s Medical Centre, Guangzhou Medical University, No.9, Jinsui Road, Guangzhou, 510623 Guangdong China; 2 School of Public Health, Guangzhou Medical University, Guangzhou, 511436 China; 3 Department of Gynaecology and Obstetrics, Guangzhou Women and Children’s Medical Centre, Guangzhou Medical University, Guangzhou, 510623 China; 4 Clinical Laboratory, Houjie Hospital of Guangdong Medical University, HeTian Road, Dongguan, 523945 Guangdong China

**Keywords:** Hypertensive disorder of pregnancy, Label-free LC-MS/MS, Complement system, Clusterin, Trophoblast

## Abstract

**Introduction:**

Hypertension disorder of pregnancy (HDP) is one of the leading causes of maternal and foetal illness. The aim of the current study was to identify and verify novel serum markers for HDP.

**Methods:**

A label-free LC-MS/MS method was used to establish the serum proteomic profiles of 12 pre-HDP (before clinical diagnosis of HDP) pregnancies and verify prioritized candidates in the verification set of 48 pre-HDP pregnancies. These biomarkers were revalidated by ELISA in an independent cohort of 88 pre-HDP pregnancies. Subsequently, the candidate biomarkers were histologically analysed by immunohistochemistry, and function was evaluated in TEV-1 cells.

**Results:**

We identified 33 proteins with significantly increased abundance and 14 with decreased abundance (peptide FDR ≤ 1%, *P* < 0.05). Complement was one of the top enriched components in the pre-HDP group compared with the control group. Three complement factors (CLU, CFHR5, and CRP) were significantly increased in the three sets, of which CLU was a critical factor for the development of HDP (OR = 1.22, *P* < 0.001). When these three factors and body weight were combined, the AUC was 0.74, with a sensitivity of 0.67 and specificity of 0.68 for HDP prediction compared with normal pregnancy. In addition, inflammation-induced CLU could inhibit the invasion of TEV-1 cells.

**Conclusions:**

Complement proteins may play an essential role in the occurrence of HDP by acting on trophoblast cells. CLU may be a high-risk factor for HDP, and the models combining candidates show reasonable screening efficiency of HDP in the first half of pregnancy.

**Supplementary Information:**

The online version contains supplementary material available at 10.1186/s12958-021-00742-z.

## Introduction

Hypertensive disorder of pregnancy (HDP) is an important cause of severe acute morbidity, long-term disability and mortality among mothers and babies, affecting up to 10% of pregnancies worldwide [1, 2]. Gestational hypertension (GH) and preeclampsia (PE) are subtypes of HDP that are commonly characterized by hypertension symptoms after 20 weeks of pregnancy. PE is a more severe subtype due to proteinuria and/or liver, kidney, and nervous system damage. Although blood pressure returns to normal after delivery, mothers with HDP and their offspring are still at high risk of cardiovascular disease. HDP is associated with various maternal high-risk factors, such as antiphospholipid antibodies, previous diabetes, multiple (twin) pregnancy, and family history of HDP [3]. The pathogenesis of HDP remains complex and diverse, and no single theory can fully explain its mechanism; thus, there is no effective prediction or treatment choice in clinical practice [4].

The current consensus is that early prediction contributes to prevention, diagnosis, and clinical intervention and can improve the prognosis of HDP. Recent studies have focused on evaluating the response of soluble vascular factor levels to disease activity for early diagnosis. However, their early prediction ability is insufficient. There is still an urgent need to identify other useful biomarkers to help predict HDP [5, 6].

An increasing number of studies have highlighted that systemic inflammation and imbalance of the maternal immune system may play a critical role in the pathogenesis of HDP [7]. Our previous results have demonstrated that inflammatory dysfunction may be detected early before the clinical onset of PE and as early as the first trimester of pregnancy [8, 9]. A recent study has shown that the overexpression of complement component C5a and C5a-receptor in the placenta can lead to maternal arterial stiffness and increased blood pressure [10]. HDP is associated with an adaptive collapse of tolerogenic cells, including a shift in T cell distribution towards Th1 and Th17, away from Th2 and regulatory CD4^+^ T cell (Treg) populations [11]. For this reason, it is of great clinical importance to identify the inflammatory and immune status in maternal blood that can predict and help prevent HDP in the preclinical state. Given the phenotypic relevance and stability of serum proteins under specific conditions, Geyer et al. proposed to use serum as a viable diagnostic biomarker source [12]. Serum contains a wide range of proteins that can act as biological signals for physiological states during homeostasis or perturbation. Recent advances in proteomics enable comprehensive evaluations of low- and high-abundance proteins in various body fluids [13, 14].

Clusterin (CLU) is a stress-activated, ATP-independent chaperone molecule that is usually secreted from cells. CLU is a new pleiotropic factor potentially involved in stimulating inflammatory cytokines such as Interleukin (IL6) and regulation of lipid metabolism, cell differentiation, cell invasion and tissue remodeling [15, 16]. The occurrence of HDP is related to insufficient trophoblast cell invasion and excessive inflammation. In this context, CLU may involve in the development of HDP.

In the current study, we used a label-free nano LC-MS/MS method to characterize serum proteomic profiles of pre-HDP (before 20 weeks of gestation) and healthy pregnant women. We also tried to understand whether these potential markers have predictive significance for HDP when used alone or in combination and to explore the role of CLU in the development of HDP.

## Materials and methods

### Study participants

This study included participants from two independent cohorts. Participants from Guangzhou Women and Children’s Medical Center (Guangzhou, China) were recruited from November 2014 to April 2017. Furthermore, participants from the Maternal & Child Health Hospital of Foshan (Foshan, China) cohort were recruited from Jul 2017 to Jun 2019. The Ethics Committee of Guangzhou Women and Children’s Medical Center and Maternal & Child Health Hospital of Foshan approved all aspects of this study (Ethics number: 2018030306). Written informed consent was obtained from all subjects. Two experienced obstetricians and gynaecologists assessed HDP and healthy pregnancies in the cohorts.

The definition of HDP by the American College of Obstetricians and Gynaecologists (ACOG) [4] includes GH (new-onset systolic blood pressure > 140 mmHg or diastolic blood pressure > 90 mmHg without significant proteinuria after 20 weeks of pregnancy) and PE (new-onset hypertension with proteinuria > 0.3 g/day or with other maternal organ dysfunction detailed in the Data supplement). Cases with a history of hypertension, nephropathy, or other diseases leading to elevated blood pressure before the 20th week of pregnancy were excluded. Furthermore, patients with primary abnormal lipid metabolism, multiple pregnancies, miscarriage, stillbirth, foetal malformations, gestational diabetes, pregnancy with thyroid disease, and pregnancy with liver and kidney disease were also excluded.

According to the inclusion and exclusion criteria, women who later developed HDP (pre-HDP) and 1:1-matched normal pregnancies (matched for maternal age, gestational age at sample collection, and sample storage time) were enrolled in this study (Fig. S1). The cases from Guangzhou Women and Children’s Medical Center were randomly divided into the screening set (12 HDP and 12 control cases) and development set (22 pre-GH, 26 pre-PE, and 48 control cases) to establish a prediction model. Cases from the Maternal & Child Health Hospital of Foshan (29 pre-GH, 59 pre-PE, and 88 control cases) were used to validate the model.

#### Serum and peptide processing

Fresh venous blood samples (2 mL) were collected from the participants before the 20th week of gestation and then centrifuged (1200×*g* for 10 min at 18 °C). Peptides were prepared following a previous protocol [13]. Briefly, peptides were stored at − 80 °C after removal of high-abundance proteins, reduction, alkylation, trypsin digestion and removal of ions.

#### Serum proteomic analysis

*LC-MS analysis.* In the two sets, serum C proteomics was analysed in triplicate using quantification label-free high-pressure liquid chromatography combined with mass spectrometry nano-LC-MS/MS (Thermo Fisher Scientific, Waltham, MA, USA). For each run, 1 μg of the digest was injected onto a 100 μm i.d. × 100 mm reverse-phase C18 BEH column with 1.7 μm particles and a 300 A pore size using a nano Acquity system (Waters, Milford, MA, USA). The chromatographic solvents were water (A) and acetonitrile (B), both with 0.1% formic acid. Peptides were eluted from the column over a gradient of 3–35% B (130 min). At 140 min, the gradient was increased to 95% B and held for 10 min. At 160 min, the gradient returned to 3% to re-equilibrate the column for the next injection. A linear gradient blank was run for 50 min between samples to prevent sample carryover. A quality control sample was tested after every 10th experimental sample. Data-dependent MS/MS-analysed peptides were eluted from the column on label-free nano LC-MS/MS. The top-15 method was used for data collection. Briefly, the instrument was set up as follows: the resolution of the MS scan was set to 70,000, and the resolution of the data-dependent MS/MS scan was set to 17,500 to improve speed. The MS AGC target was set to 106 counts, while the MS/MS AGC target was set to 105. The MS scan range was 300–2000 m/z. MS scans were recorded in profile mode and MS/MS in centroid mode to reduce the data file size. Dynamic exclusion was set to a repeat count of 1 and a duration of 25 s.

#### Protein identification

The raw MS files were processed using MaxQuant (version 1.5.6.0). The human protein sequence database (UniProt_human_2016_09) was downloaded from UNIPROT. This database and its reverse decoy were then searched against using MaxQuant. Trypsin was set as a specific enzyme with up to two missing cleavages. Oxidation (M) and acetyl (protein N-term) were considered variable modifications, and carbamidomethyl (C) was set as the fixed modification. The minimum and maximum peptide lengths were seven and 4600, respectively. Both the peptide and protein false discovery rates (FDRs) were < 0.01.

### Bioinformatics

A volcano plot and heatmap were used to evaluate the proteomic profile cluster and functional analysis. Heatmap was set considering *P*-values between the two groups and the Ward’s methodology as clustering algorithm. When the fold change (FC) was > 1.2 or < 0.8 and the P-value was < 0.05, serum protein was considered the protein of interest. For differentially expressed proteins, functional annotation was performed using the DAVID and Gene Ontology (GO) databases. Metascape software (http://metascape.org/) was used to perform protein-protein interaction (PPI) and pathway clustering [17].

### Candidate biomarker evaluation

Human CLU (Abcam, Cambridge, UK) and complement factor H-related protein 5 (CFHR5, Raybiotech, Norcross, GA, USA) immunoassays were used to determine the levels of CLU and CFHR5 in another group of pregnant women (second validation set) before 8–20 weeks of gestation (*n* = 88). The procedure was performed according to each manufacturer’s instructions. Serum C-reactive protein (CRP) was detected by an i7600 automatic biochemistry analyser (Hitachi, Japan).

### Analysis of prediction efficiency

First, the performance of the verified candidate proteins was evaluated by calculating the ROC curves of the individual and combined proteins in each verification cohort. ROC analysis was performed by setting diagnostic HDP (after 20 weeks) as a positive test. When performing combination marker analysis, the probability is calculated by logistic regression, and the ROC curve is combined to evaluate the predictive ability of the model. The coordinates of the curve were then output to estimate the potential cut-off value.

### Immunohistochemistry

Placental tissues of women with PE and normal pregnancy were obtained immediately after delivery. Following collection, the tissue was extensively washed in PBS and fixed in 4% neutral buffered formalin for 24 h. The paraffin-embedded placental sections (3 μm) were deparaffinized through xylene and rehydrated by graded ethanol. Antigen retrieval was performed using EDTA antigen retrieval solution (pH 8.0) and microwave heating (15 min). Then, 3% hydrogen peroxide was used to quench endogenous peroxidase activity, and the slides were blocked with 5% BSA. Afterward, they were stained using anti-CLU antibodies (1:100, Affinity, USA) and incubated overnight at 4 °C. The Dako REAL™ EnVision™ Detection System, Peroxidase/DAB+, and Rb/Mo (K5007, Dako Denmark A/S, Denmark) were then utilized to detect the bound antibody. A total of six fields were randomly selected and measured semi-quantitatively using ImageJ software (National Institutes of Health, Bethesda, MD).

### Quantitative real-time PCR

Placental tissue was ground in liquid nitrogen, and then total RNA was extracted with TRIzol reagent (Invitrogen, Waltham, MA) following the manufacturer’s instructions. Microspectrophotometry and gel electrophoresis were used to test the purity and integrity of RNA. For PCR of mRNA, RNA was reverse transcribed using HiScript III RT SuperMix (Vazyme, Nanjing, China). qPCR was performed using ChamQ SYBR qPCR Master Mix (Vazyme, Nanjing, China) on the LC480 system (Roche, La Jolla, CA, USA). The specific primer was listed in the data supplementary. The relative expression slevels were calculated using the 2^(−△△CT)^ method.

### Cell culture

TEV-1, a human extravillous trophoblast cell line, was a gift from The University of Hong Kong and The Chinese University of Hong Kong. Cells were cultured in Dulbecco’s modified Eagle’s medium: DMEM/F12 supplemented with 10% foetal bovine serum and 1% penicillin/streptomycin (Gibco; Thermo Fisher Scientific, Waltham, MA, USA) in an incubator containing 5% CO_2_ at 37 °C.

### Western blot

EVT-1 cells were treated with LPS for 24 h, whole lysates were prepared by direct lysis in RIPA buffer with PMSF (Beyotime, Beijing, China). Electrophoresis processes were performed as previously described [18]. Primary antibodies of CLU (DF6421, Affinity, Cincinnati, OH, USA) and GAPDH (AF7021, Affinity, Cincinnati, OH, USA) were used in this analysis. Antigens were revealed using a chemiluminescence assay (Affinity, Cincinnati, OH, USA). Quantification of bands was achieved by densitometry using the Chemiluminescence Image analysis system (GE Healthcare, BI600, Boston, MA, USA).

### Matrigel transwell assay

TEV-1 cells were starved for 12 h, digested and resuspended in serum-free DMEM/F12 (1 × 10^5^ cell/ml). Transwell chambers for the invasion assay were precoated with Matrigel. Two hundred microlitres of cell mix was added to the upper chamber, and then 500 μL DMEM/F12 medium supplemented with 20% FBS was added to the lower chamber. In addition, the lower chamber contained recombinant human CLU (R&D Systems, Minneapolis, Minnesota, USA) at final concentrations of 0, 5, 10, 20, 50, and 100 ng/ml. After 24 h, cells were fixed with 4% paraformaldehyde for 20 min and stained with 0.1% crystal violet for 10 min, and then images were captured with a microscope. The level of cell invasion was quantified by averaging the total number of cells in three random fields. The experiment was repeated three times.

### Statistical analysis

The chi-square test was used to compare categorical variables between patients and controls. Independent *t*-tests were used to compare the differences between continuous variables. Differentially expressed proteins were determined using fold change> 1.2 and *P*-value< 0.05. A volcano plot was generated using R software (version 3.6.2) with the ggplot2 package, and the PPI network was constructed using the STRING database (https://string-db.org) and Cytoscape software (version 3.7.2). Logistic regression was used to estimate the odds ratio (OR), and the ROC curve was used to evaluate the model prediction capability. To compare quantitative variables of serum proteins and mRNA expression of placenta, Student’s *t*-test and one-way ANOVA were used where appropriate. Data were analysed with SPSS (version 22.0; IBM Inc., city NY, USA). A two-tailed *P* < 0.05 was considered statistically significant.

## Results

### Clinical characteristics

The features of the study participants are presented in Table 1. The demographics, medical history, and new-borns of healthy and pre-HDP pregnancies were compared. Women with an elevated body mass index (BMI) before 20 weeks of gestation were more likely to develop HDP, and there was foetal growth restriction in the HDP group (*P* < 0.05).

**Table 1:** Characteristics of pre-HDP patients and healthy pregnancies

Factors	The screening and validation set		
	Control (***n*** = 148)	Pre-HDP (***n*** = 148)	***P***
	N (%)	N (%)	
**Demographic characteristics**			
Han nationality, n (%)	145 (97.97)	145 (97.97)	1.00
Maternal age (years)	30.84 ± 4.46	30.99 ± 4.69	0.83
Weight (kg)	64.71 ± 9.16	72.12 ± 11.44	< 0.001
BMI (kg/m^2^)	25.37 ± 2.47	28.49 ± 3.98	< 0.001
Diastolic (≤ 20 wk)	73.54 ± 12.94	87.9 ± 12.96	< 0.001
Systolic (≤ 20 wk)	117.4 ± 17.63	138.39 ± 15.60	< 0.001
GA at sample collection	14.27 ± 4.80	14.36 ± 4.64	0.917
GA at delivery	39.02 ± 0.95	37.45 ± 2.39	< 0.001
**Medical history**			
Anaemia n (%)	14 (10.22)	24 (18.46)	0.054
Family history of DM, n (%)	2 (1.47)	6 (4.35)	0.16
Family history of HDP, n (%)	1 (0.74)	2 (1.45)	0.57
**Past obstetric history**			
Gravidity ≥2, n (%)	93 (62.84)	95 (64.19)	0.81
History of term delivery ≥1, n (%)	117 (79.05)	106 (71.62)	0.14
Spontaneous miscarriage ≥1, n (%)	21 (14.19)	26 (17.57)	0.43

*GA* gestational age, *BMI* body mass index, *DBP* diastolic blood pressure, *SBP* systolic blood pressure, *DM* diabetes disease, *HDP* hypertension disorder of pregnancy. Data are presented as the mean ± SD (standard deviation) and analysed by Student’s *t*-test. Data are presented as n (%) and were analysed using a chi-square test or Fisher’s exact test. Women who developed HDP and women who had a healthy pregnancy were matched by age and gestational week

### Screening set

**Fig. 1:**
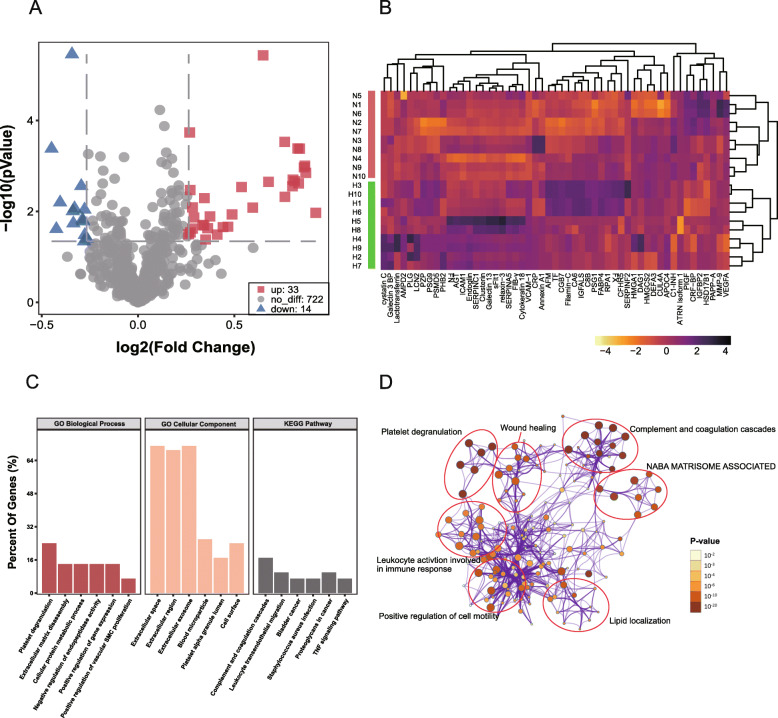
Protein profiles differ significantly between pre-HDP and normal pregnancies (*n* = 12). **a**, A volcano plot was used to analyse differentially expressed proteins. Red nodes represent upregulated proteins, and blue nodes represent downregulated proteins. Fold change> 1.2 and *P*-value< 0.05. **b**, Log_2_-transformed relative protein expression heatmap of all proteins profiled in the screening set. Green indicates HDP patients, and purple indicates healthy controls. Pearson correlation was used for clustering of proteins and Spearman’s correlation was used for samples. **c**, GO and KEGG enrichment of proteins included in the screening set (*n* = 47). Bands represent the top 6 enriched component terms. **d**, Pathway cluster analysis in Metascape software. Node size represents the number of pathway-enriched genes, and shade of colour represents the size of the adjusted *P*-value

Based on label-free LC-MS/MS, we identified a total of 769 serum proteins in the screening set (*n* = 24); among them, 33 proteins were upregulated, and 14 were downregulated (Fig. 1a). Pearson correlation analysis was used in the heatmap to cluster the proteins and samples separately, and this showed that these 47 proteins could be divided into four categories, which could distinguish the disease group from the control group (Fig. 1b). The biological process, cellular component, and KEGG (top 6) analyses showed that inflammation activation and the complement system might play a key role in HDP development (Fig. 1c). To further capture the relationships between the terms, a subset of enriched terms was selected and rendered as a network plot, where terms with a similarity > 0.3 were connected by edges. The terms with the best *p*-value were selected from 20 clusters, each cluster was limited to no more than 15 terms, and the total did not exceed 250 terms. Pathway cluster analysis further indicated the importance of complement system activation, which involved platelet degranulation, wound healing, and inflammatory responses (Fig. 1d).

### First verification phase

**Fig. 2:**
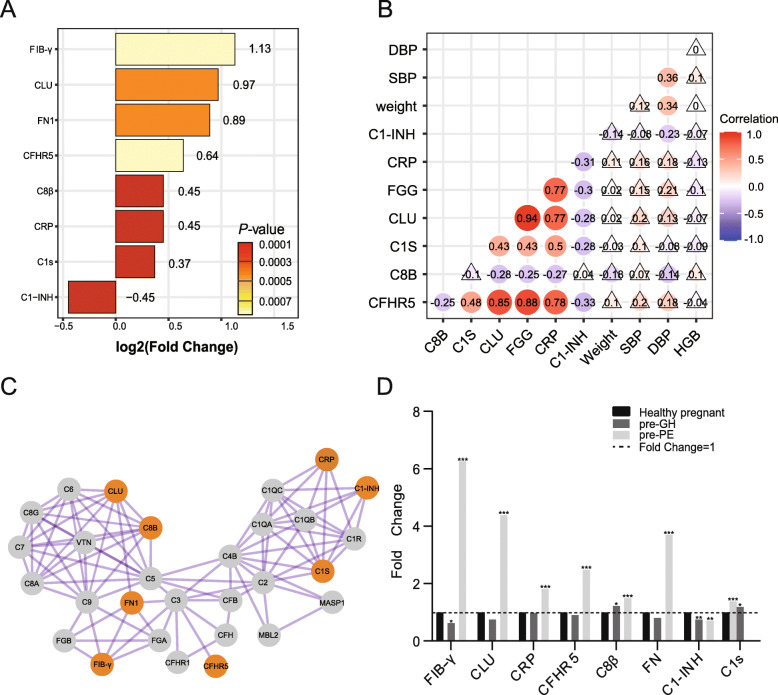
Differences in complement protein levels between pre-HDP and normal pregnancy (*n* = 48). **a**, Eight differentially expressed complement proteins in the second validation set. The number after each bar represents log2 (fold change), and the bar colour represents the size of the *P*-value. **b**, Pearson’s correlation revealed the correlation of eight complement proteins and four clinical factors. The lighter colour represents the size of the correlation coefficient, and the triangle means the *P*-value> 0.05. SBP = systolic blood pressure, DBP = diastolic blood pressure, HGB = haemoglobin. **c**, PPI network of complement-related proteins. The orange nodes represent the eight complement proteins that were screened. **d**, Protein levels are heterogeneous between pre-GH and pre-PE

In the first verification phase, the sample size was expanded, and the same methodology was repeated with a focus on the complement proteins. The verification data were consistent with those of the screening phase by 93.5% (Table. S2). In the first verification phase, a total of eight proteins associated with the complement system (FC > 1.2; *P* < 0.05), including CLU, fibrinogen gamma chain (FIB-γ), complement factor H-related protein 5 (CFHR5), complement C8 beta chain (C8β), complement C1s (C1s), complement C1 inhibitor (C1-INH), fibronectin (FN), and C-reactive protein (CRP), were significantly different (Fig. 2a).

The CLU level was significantly increased in pre-HDP pregnancies (FC = 1.96, *P* = 0.0004) and was positively correlated with disease severity (FC was 0.75 for GH vs. 4.39 for PE). Pearson’s correlation demonstrated that CLU had a strong positive relationship with FIB-γ (*r* = 0.937) and CFHR5 (*r* = 0.847), which indicated that their role in pre-HDP might be highly relevant (Fig. 2a). Besides, we evaluated the relationship between these clinical features and candidate markers because there are large differences in patient’s baseline weight, blood pressure, and anaemia (Table 1 and Table 2). Bodyweight, systolic blood pressure (SBP), diastolic blood pressure (DBP), and haemoglobin concentration (HGB, represent the degree of anaemia) have no-significance relationships with most of these eight proteins, and C1-INH was negatively correlated with DBP (Fig. 2b). Via PPI analysis, CLU was a critical factor for the development of HDP and had a function in the membrane attack complex (C5b-9, MAC), while FIB-γ and CFHR5 showed a correlation with C3 (Fig. 2c). C8β, C1s, and C1-INH showed a low correlation with other proteins. We further analysed whether these proteins were expressed differently in pre-GH and pre-PE pregnancies. The CLU, FIB-γ, CFHR5, FN, and CRP levels in pre-PE pregnancies were higher than those in the pre-GH group (Fig. 2d).

**Table 2:** Maternal adverse outcomes of pre-HDP patients and healthy pregnancies

Factors	The screening and validation set		
	Control (***n*** = 148)	Pre-HDP (***n*** = 148)	***P***
	N (%)	N (%)	
**Maternal adverse outcomes**			
Parturient at admission, n (%)	126 (86.7)	102 (73.2)	0.001
PROM, n (%)	32 (24.06)	27 (19.57)	0.97
Umbilical cord around neck, n (%)	26 (19.12)	21 (15.79)	0.47
Caesarean section, n (%)	6 (4.42)	61 (45.86)	< 0.001
Postpartum haemorrhage, n (%)	2 (1.47)	0 (0)	0.16
Oedema of lower limbs, n (%)	3 (2.17)	18 (13.53)	0.08
Soreness of waist, n (%)	0 (0)	7 (5.26)	/
Cramps, n (%)	0 (0)	0 (0)	/
**Infant**			
Birth weight (g)	3196.15 ± 320.21	2823.69 ± 680.40	< 0.001
**Paternal condition**			
Paternal age (years)	31.72 ± 6.06	31.52 ± 5.83	0.81

*PROM* Premature rupture of membranes. Data are presented as the mean ± SD (standard deviation) and analysed by Student’s *t*-test. Data are presented as n (%) and were analysed using a chi-square test or Fisher’s exact test

### Model construction

**Fig. 3:**
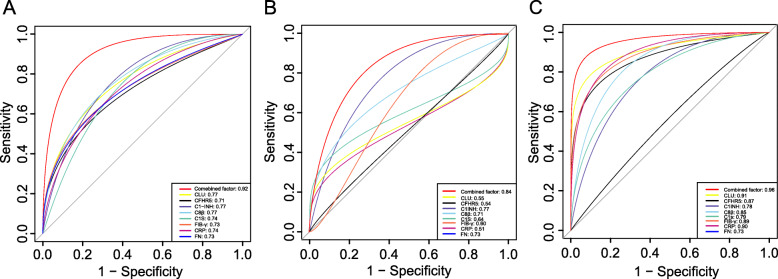
A, Receiver operating curve (ROC) of individual and combined markers for HDP by LC-MS/MS (*n* = 48). B, ROC curve of markers for GH prediction (*n* = 22). C, ROC curve of markers for PE prediction (*n* = 26). The area under the curve (AUC) for each curve is listed in the lower right corner. HDP: hypertension disorder of pregnancy, GH: gestational hypertension, PE: preeclampsia

In the first verification phase, the capability of models of these eight proteins to predict HDP was determined by ROC analysis (Fig. 3). The results showed that some of the proteins have a potential value to discriminate before clinical symptoms occur. For HDP prediction, the AUCs were C8β (0.77), CLU (0.77), CFHR5 (0.71), CRP (0.74), FIB-γ (0.73), C1s (0.74), FN (0.73), C1-INH (0.77) with *P* < 0.05 for all these values. Some clinical characteristics also showed potential predictive ability, such as body weight (0.68), SBP (0.69), DBP (0.75) and HGB (0.58), and their ROC curves were concluded in Fig. S3.

A predictive model was then developed through logistic regression based on the clinical characteristics and the candidate markers. When CLU, CFHR5, C8β, CRP, and body weight were used in combination, the capability of HDP prediction was the highest, which AUC increase to 0.92 (95% CI 0.83–0.99) with the sensitivity of 0.80, and the specificity of 0.88 (Fig. 3a). Moreover, in the analysis of HDP subtype, the AUC of combined CLU, CFHR5, C8β, and CRP for GH prediction was 0.84 (95% CI 0.63–0.92) with a sensitivity of 0.78 and specificity of 0.89 (Fig. 3b), and for PE, it was 0.96 (95% CI 0.94–1.00) with a sensitivity of 0.87 and specificity of 0.92 (Fig. 3c).

### Model validation

**Fig. 4:**
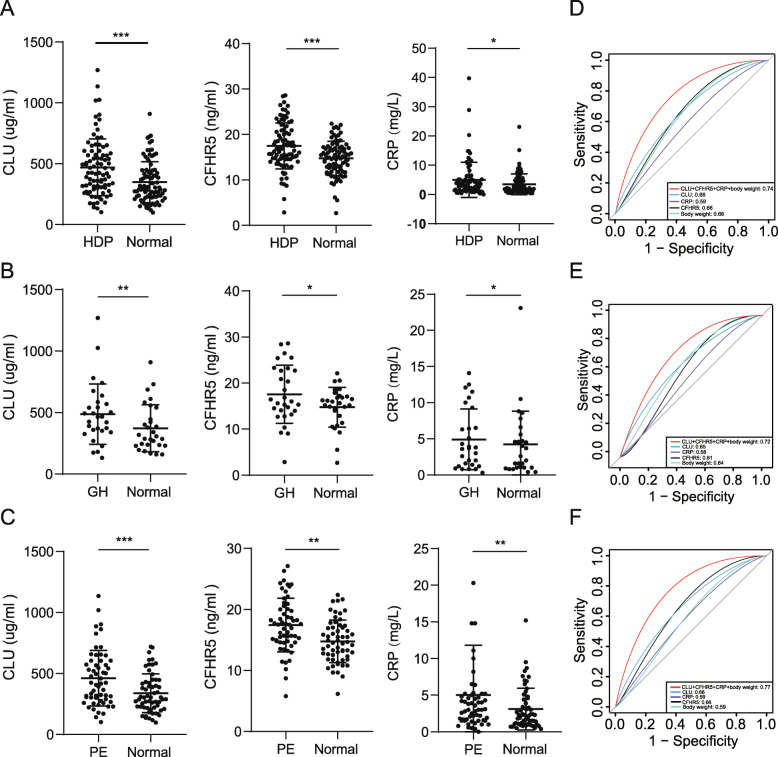
**a**-**c**, ELISA analysis of serum levels of CLU, CFHR5, and CRP before 20 weeks gestation in women who experienced an uncomplicated pregnancy and women who later developed HDP (88 HDP and 88 normal pregnancy cases). **b**, Serum levels of CLU, CFHR5, and CRP in GH and normal pregnancies (*n* = 22). **c**, Serum levels of CLU, CFHR5, and CRP in PE and normal pregnancies (*n* = 26). Data are presented as the mean ± SD and were analysed with a paired t-test. **d**-**f**, ROC curve of HDP prediction. **d**, ROC curve of HDP prediction. **e**, ROC curve of GH prediction. **f**, ROC curve of PE prediction. The area under the curve (AUC) for each curve is listed in the lower right corner. *P < 0.05, **P < 0.01, ***P < 0.001

In the second verification phase, the efficacy of this model was validated in another independent cohort (including 29 pre-GH, 59 pre-PE, and 88 normal pregnancies). ELISA kits were used due to their high repeatability and specificity. Serum CLU, CFHR5, and CRP increased in the pre-HDP groups compared with the normal pregnancies (Fig. 4a-c). Compared to those in normal pregnancies, CLU (469.71 ± 211.53 vs. 349.49 ± 169.59 μg/ml, OR = 1.22, *P* < 0.001), CFHR5 (17.49 ± 4.65 vs. 14.78 ± 3.76 ng/ml, OR = 1.13, *P* < 0.001), and CRP (4.99 ± 6.03 vs. 3.48 ± 3.51 mg/L, OR = 1.27, *P* = 0.04) were increased in pre-HDP pregnancies. The levels of CLU (416.54 ± 236.07 vs. 337.59 ± 166.76 μg/ml, *P* < 0.001) and CFHR5 (17.26 ± 5.00 vs. 14.11 ± 3.70 ng/ml, *P* < 0.001) in pre-GH pregnancies were higher than those in normal pregnancies, but there was no difference in CRP (3.40 ± 6.07 vs. 2.90 ± 3.25 mg/L, *P* = 0.012). In addition, the levels of CLU (492.76 ± 232.95 vs. 354.49 ± 170.44 μg/ml, *P* < 0.001), CFHR5 (17.52 ± 5.08 vs. 15.07 ± 3.77 ng/ml, *P* < 0.001), and CRP (5.82 ± 6.03 vs. 3.68 ± 3.51 mg/L, *P* = 0.039) were higher in pre-PE pregnancies than in normal pregnancies.

The AUC for the HDP prediction model of CLU combined with CFHR5, CRP and body weight was 0.74 with a sensitivity of 0.67 and specificity of 0.68 (Fig. 4d). For GH prediction, the AUC was 0.72 with a sensitivity of 0.60 and specificity of 0.72 (Fig. 4e). For PE, the AUC was 0.77 with a sensitivity of 0.79 and specificity of 0.73 for PE prediction (Fig. 4f).

### Localization and expression of CLU in the placenta

**Fig. 5:**
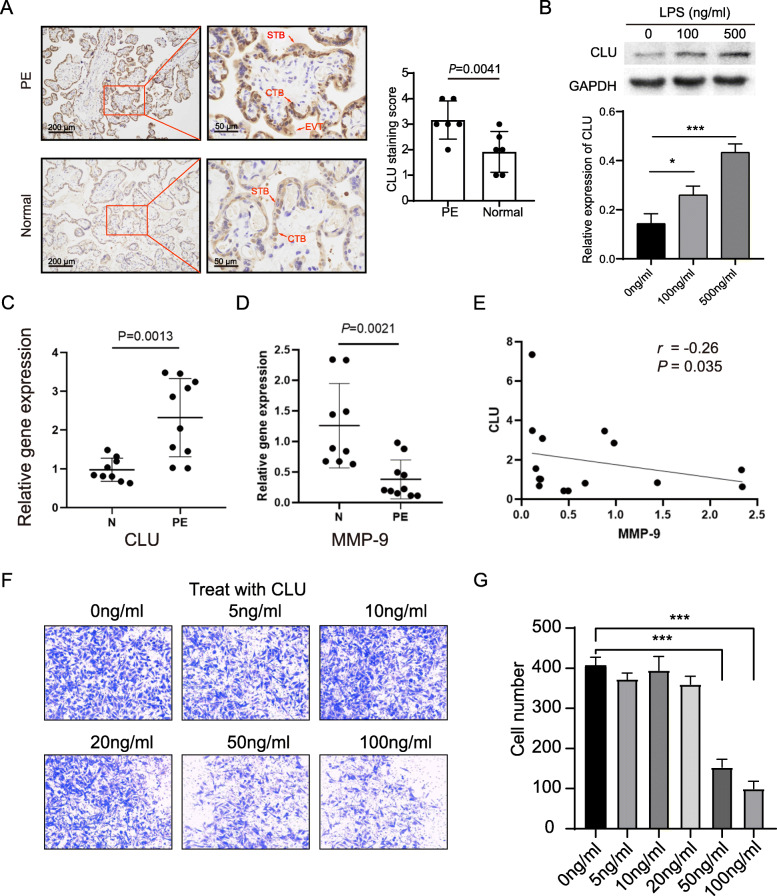
CLU is upregulated in the placenta from PE deliveries. **a**, Fixed placental tissue sections were used for immunohistochemical analysis. Student’s t-test, *n* = 6. **b**, LPS stimulation leads to increased expression of CLU in TEV-1 cells. The upper part shows a representative western blot band and below is the relative quantification after normalization. One-way ANOVA, *n* = 3. **c**, Total placental RNA was extracted for relative quantification of CLU mRNA levels by qRT-PCR (*n* = 19). **d**, Total placental RNA was extracted for relative quantification of MMP9 mRNA levels (*n* = 19). **e**, Placental CLU gene expression was negatively correlated with MMP9 via Pearson’s correlation. **f**, CLU inhibits TEV-1 cell invasion. The left panel is a representative micrograph of the Matrigel Transwell assay treated with CLU. The right panel represents quantitative results of cell invasion, and data are presented as the mean ± SD and were analysed by one-way ANOVA, *n* = 3. *P < 0.05, **P < 0.01, ***P < 0.001. PE = Preeclampsia, N=Normal pregnancy, CLU=Clusterin, MMP9 = Matrix metalloproteinases 9

CLU is immunolocalized extravillous trophoblasts (EVTs), syncytiotrophoblasts (STBs), and cytotrophoblasts (CTBs) of the placenta (Fig. 5a). The CLU immunostaining intensity was higher than that in normal pregnancies, while CLU mRNA expression was higher in PE placentas than in healthy controls (Fig. 5b).

### LPS promotes CLU expression

The CLU expression of TEV-1 cells was significantly upregulated following treatment with 100 ng/ml and 500 ng/ml LPS (*P* = 0.001) (Fig. 5b). And CLU mRNA level is positively correlated with LPS concentration (Fig. S2).

### CLU inhibits invasion of EVT

The expression of MMP9 mRNA was downregulated in the placentas of pregnant women with PE and negatively correlated with CLU mRNA (Fig. 5c, d). TEV-1 cell invasion was inhibited by treatment with 50 ng/ml and 100 ng/ml CLU, as determined by the invasion assay (*P* < 0.001, Fig. 5e).

## Discussion

In the current study, the difference in serum proteins between pre-HDP and normal pregnancies was identified by a label-free nano LC-MS/MS method, and 47 differential proteins were found. Subsequently, a novel prediction model for HDP was constructed, and it was validated in another independent cohort. Additionally, in in vitro assays, CLU inhibited the invasion of TEV-1 cells.

Increasing studies have highlighted that complement system disorders are related to the occurrence and development of HDP and that complement molecules may be potential markers for PE screening and diagnosis [11]. The results of the current study also showed that in pre-HDP, there is disorder among components of the complement system that are involved in the complement classical pathway (CRP and C1s), complement membrane attack (C8β), and complement regulation (CLU, CFHR5, and C1-INH). To the best of our knowledge, this is the first study to find that pre-HDP pregnancies have elevated levels of CFHR5 and C8β. Previous studies have found that complement factors Bb and C5a are negatively correlated with placental growth factor (PlGF), leading to incomplete spiral artery remodeling and endothelial dysfunction [19, 20]. Kim and Kolla also found that C1s and FIB-γ levels were increased in PE [21, 22]. Researchers believe that the increase in placenta-derived debris and apoptotic granules in the circulation of HDP pregnancies is the leading cause of the activation of the complement classical pathway and alternative pathway [23]. Excessive dysregulation of the complement system may lead to the insufficient clearance of apoptotic granules and placenta-derived debris and result in excessive inflammation, which may lead to the typical symptoms of HDP (proteinuria and new-onset hypertension) [11]. Mutations in the complement inhibitor gene can also cause HELLP syndrome (a severe subtype of HDP) [23–25]. Moreover, Burwick and colleagues successfully treated patients with HELLP syndrome with eculizumab (a C5 targeted inhibitor), which showed that suppressing complement activation could improve HDP symptoms [26].

Disorder of complement regulatory factors (CLU, CFHR5, and C1-INH) may be associated with excessive inflammation and visceral organ injury in mothers with HDP. CFHR5, an analogue of complement factor H, can enhance local AP activation via interference with the C-inhibiting function of factor H in the cell membrane [27, 28]. Existing studies have not found an association with pregnancy-related diseases, but CFHR5 is positively associated with kidney injury. CFHR5 mutation was considered the cause of atypical haemolytic uraemic syndrome [29]. Through the mechanism of kidney injury, we speculate that elevated CFHR5 causes excessive AP activation, which leads to vascular endothelial damage and the release of inflammatory factors, ultimately resulting in PE. On the other hand, this suggests that renal impairment may occur before blood pressure increases.

Our team considered that CLU might be a critical biomarker for the prediction of the development of HDP. CLU is a secreted glycoprotein, founding in various body fluids, which has multiple functions, including regulating apoptosis, translocating lipids, inhibiting complement, regulating immunity, and cell invasion [30]. Some literature highlighted that CLU was associated with pregnancy diseases, such as intrauterine growth restriction, recurrent pregnancy loss, and small gestational age [31–33]. There are some intersecting pathophysiological mechanisms between HDP and intrauterine growth restriction and recurrent miscarriage involved in placental implantation and development dysfunction [34]. However, the role of CLU in placental development and trophoblast differentiation is still unclear. It is currently recognized that insufficient trophoblast invasion and lack of differentiation are part of the pathogenesis of HDP [35]. Previous research has reported that CLU could regulate the proliferation and invasion of vascular smooth muscle and tumor [15, 36, 37]. The current study shows that CLU characteristics are interesting in the pathogenesis of HDP. First, the fact that serum CLU was positively correlated with the severity of HDP, and it was mainly localized to CTBs at the HDP placenta, suggests that it is important in the development of HDP and the differentiation of trophoblast cells. Second, we demonstrated that the invasion ability of EVTs was reduced by treating CLU, which expression increased was related to LPS treatment. Therefore, inflammation-induced CLU could inhibit EVT’s invasion, which further may cause insufficient spiral artery remodeling and placental ischemia. Eventually, it will lead to increased maternal oxidative stress and cause systemic symptoms. Besides, compared with other candidates in this work, although the CLU increase is not the highest, it plays an important role in regulating oxidative stress and cell apoptosis [30]. The occurrence and development of HDP involved excessive oxidative stress and apoptosis too [35]. So, we think it is interesting to study CLU’s effect on oxidative stress and apoptosis in HDP, and these hypotheses are worthy of further exploration.

We found that LPS-induced inflammation increases CLU expression, indicating CLU elevation is a downstream response induced by excessive inflammation. In HDP, the inflammatory response is inflated due to aberrant activation of innate immune cells and imbalanced differentiation of T-helper cell subsets [11, 38]. In this study, serum FN, FIB-γ and CRP were more abundant in HDP pregnancies than normal pregnancies. Previous studies have found that pre-PE pregnancies have more abundant FN, FIB-γ and CRP levels involved in excessive inflammation and organ damage [39–42]. CLU level may involve inflammation-induced organ damage because it’s highly correlated with inflammation markers (FN, FIB-γ, and CRP).

Evaluation of the performance of markers indicated that the combination of multiple factors can provide a better prediction model than any of the individual proteins alone for predicting HDP. In the screening cohort, ROC analysis showed that individual panels comprising C8β, CLU, CFHR5, CRP, FIB-γ, C1s, FN, and C1-INH performed well in predicting GH and PE. Logistic regression was used to select possible risk factors from clinical characteristics and candidate markers to develop a predictive model. The highest predictive capability model was then generated for HDP (CLU, CFHR5, C8β, CRP, and body weight). This model was subsequently verified in another cohort. Compared with those in normal pregnancies, these three proteins’ levels increased in pre-HDP pregnancies with the ELISA method. The combination of CLU, CRP, CFHR5, and body weight for prediction of HDP has an AUC of 0.74, a sensitivity of 0.67, and a specificity of 0.68. Compared with LC-MS/MS approach, although the AUC decreases in the ELISA method, the validation model still has considerable prediction efficiency. Especially for PE, the model has a higher prediction efficiency, which is important for distinguish PE from GH. And the model is independent of the clinical characteristics, including blood pressure and body weight, which was not related to elevated candidate biomarkers. Due to the exhaustion of specimens, only CLU, CRP, and CFHR5 were verified; otherwise, this model would be more reliable if C8β was also added.

This study has some limitations. Compared with the clinical environment, the small sample size may cause differences in sensitivity and specificity. Thus, we will expand the research object in the next study and conduct a multicentre study to further improve the model. The role of CLU in HDP development needs to be further verified in appropriate in vitro and in vivo models.

## Conclusions

In summary, we consider that there are dysregulated classical pathways and alternative pathways of the complement system, leading to excessive inflammation and endothelial damage, which is associated with the development of HDP. Moreover, CLU can inhibit EVT cell invasion in vitro. The combination of CLU, CFHR5, C8β, CRP, and body weight might improve the early prediction of HDP.

## Supplementary Information

**Additional file 1.**

## Data Availability

All data are included in the article.

## Abbreviations

HDP: Hypertensive disorder of pregnancy
GH: Gestational hypertension
PE: Preeclampsia
CLU: Clusterin
MMP9: Matrix metalloproteinases 9
GAPDH: Glyceraldehyde-3-phosphate dehydrogenase
OR: Odds ratio
BMI: Body Mass Index
FIB-γ: Fibrinogen gamma chain
CFHR5: Complement factor H-related protein 5
C8β: Complement C8 beta chain
C1s: Complement C1s
C1-INH: Complement C1 inhibitor
FN: Fibronectin
CRP: C-reactive protein
PPI: Protein-protein interaction
MAC: Membrane attack complex
STB: Syncytiotrophoblast
PIGF: Placental growth factor
EVT: Extravillous trophoblast
ROC: Receiver operating characteristic curve
AUC: Area Under Curve
