# Supplementary material for: Serum complement proteomics reveal biomarkers for hypertension disorder of pregnancy and the potential role of Clusterin
Source: Reprod Biol Endocrinol. 2021 Apr 19;19:56. doi: 10.1186/s12958-021-00742-z (PMC8054419; doi:10.1186/s12958-021-00742-z)
Supplement: Supplementary file 1 — Additional file 1. [file 12958_2021_742_MOESM1_ESM.docx]

Data supplementary

Detailed diagnosis and inclusion and exclusion criteria

HDP is characterized by new-onset hypertension and proteinuria after the 20th week of gestation that was resolved at 6 weeks postpartum. The blood pressure of the patients was documented on two separate occasions at least 6 h apart. For the control group, participants exhibited normal blood pressure without an excess of protein in the urine, pregnancy complications, or other fetal malformations. For the subjects who subsequently developed HDP, we excluded women who had multiple pregnancies, gynecological disease, gestational diabetes mellitus, pre-gestational type 1 and type 2 diabetes, chronic hypertension, and cardiovascular, liver, or kidney diseases. HDP is defined as a systolic blood pressure 140 mm Hg or more or a diastolic blood pressure of 90 mm Hg or more, or both, on two occasions at least 4 h apart after 20 weeks of gestation, in a woman with a previously normal blood pressure [13]. HDP is considered severe when the systolic level reaches 160 mm Hg or the diastolic level reaches 110 mm Hg, or both. HDP occurs when hypertension without proteinuria or severe features develops after 20 weeks of gestation and blood pressure levels return to normal in the postpartum period. Preeclampsia was defined as systolic blood pressure≥140 mmHg and/or the diastolic blood pressure ≥90 mmHg in a pregnant woman after 20 weeks of pregnancy, accompanied by any of the following: urine protein quantitative ≥0.3 g/24 h, or urine protein/creatinine ratio ≥0.3, or random urine protein ≥ (+) (examination method for protein quantification without conditions); no proteinuria but with any of the following organ or system involvement: heart, lung, liver, kidney and other important organs, or blood system, abnormal changes in the digestive system and nervous system, placental-fetal involvement, etc. Preeclampsia can also occur after delivery. The continuous increase in blood pressure and/or urine protein levels, or the involvement of maternal organs or the occurrence of placental-fetal complications are manifestations of the progression of preeclampsia. Pre-eclampsia pregnant women have any of the following manifestations as severe pre-eclampsia: (1) The blood pressure continues to rise and cannot be controlled: systolic blood pressure ≥160 mmHg and (or) diastolic blood pressure ≥110 mmHg; (2) Persistent headache, visual disturbance or other central nervous system abnormalities; (3) Persistent upper abdominal pain and liver subcapsular hematoma or liver rupture; (4) Abnormal aminotransferase levels: blood alanine aminotransferase (ALT) or days Increased aspartate aminotransferase (AST) level; (5) Impaired renal function: urine protein quantitative >2.0 g/24h; oliguria (24-h urine output <400 ml, or hourly urine output <17 ml), Or blood creatinine level>106 μmol/L; (6) Hypoproteinemia with ascites, pleural effusion or pericardial effusion; (7) Abnormal blood system: platelet count is continuously decreased and less than 100 × 109/L; microvascular Hemolysis, manifested by anemia, elevated blood lactate dehydrogenase (LDH) levels or jaundice; (8) heart failure; (9) pulmonary edema; (10) fetal growth restriction or oligohydramnios, intrauterine death, Placental abruption, etc.


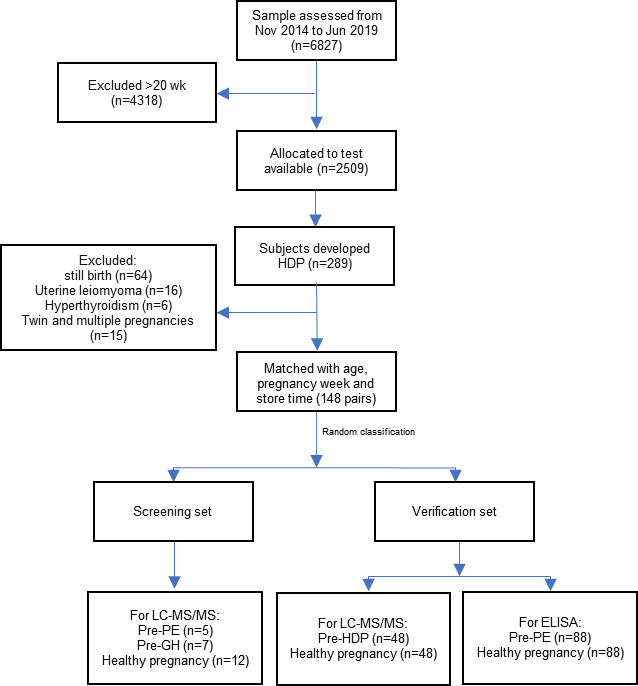
Fig. S1 Flow chart of the study design

Table S1. Primers used in this work:

| **Gene name** | **Forward primer** | **Reverse primer** |
| --- | --- | --- |
| *CLU* | 5’-GCATACCTGCATGAAGTTCTATG-3’ | 5’-GTAGAAGGGTGAGCTCTGGTTT-3’ |
| *MMP9* | 5’-CGCAGACATCGTCATCCAGT-3’ | 5’-AACCGAGTTGGAACCACGAC-3’ |
| *GAPDH* | 5’-TGTTGCCATCAATGACCCCTT-3’ | 5’-CTCCACGACGTACTCAGCG-3’ |

Table S2. Serum proteins represented in the screening phases

| UniqueID | Name | Screening set (n=12) | | Verification set (n=48) | |
| --- | --- | --- | --- | --- | --- |
|  |  | Fold Change | P-value | Fold Change | P-value |
| P02679 | FGG | 1.82 | 0.0011 | 2.19 | 0.0009 |
| P10909 | Clusterin | 1.79 | 0.0004 | 1.96 | 0.0004 |
| P09871 | C1s | 1.32 | 0.0414 | 1.29 | <0.0001 |
| Q9BXR6 | CFHR5 | 1.26 | 0.0154 | 1.56 | 0.0009 |
| P07358 | C8β | 1.22 | 0.0081 | 1.37 | 0.0001 |
| P02748 | C9 | 1.19 | 0.0046 | 1.17 | 0.03 |
| P0C0L5 | C4b | 1.16 | 0.0072 | 1.19 | 0.006 |
| P00746 | CFD | 1.14 | 0.0072 | / | / |
| P06681 | C2 | 1.13 | 0.0114 | 1.19 | 0.002 |
| P10643 | C7 | 1.10 | 0.0298 | 1.15 | 0.02 |
| P08603 | CFH | 1.07 | 0.0345 | 1.14 | 0.03 |
| Q03591 | CFHR1 | 1.05 | 0.3643 | 1.15 | 0.04 |
| P07357 | C8α | 1.04 | 0.0341 | 0.94 | 0.2 |
| P01031 | C5 | 1.02 | 0.0718 | 0.96 | 0.5 |
| P00736 | C1r | 1.01 | 0.5888 | 0.93 | 0.2 |
| P00751 | CFB | 0.99 | 0.8733 | 0.95 | 0.5 |
| P48740 | MASP-1 | 0.95 | 0.2602 | 0.83 | 0.0002 |
| P01024 | C3 | 0.94 | 0.0275 | 1.13 | 0.03 |
| P02745 | C1QA | 0.89 | 0.0438 | 0.87 | 0.03 |
| P02675 | FIB-β | 0.89 | 0.2199 | 0.85 | 0.002 |
| P02671 | FIB-α | 0.86 | 0.0957 | 0.86 | 0.006 |
| P04004 | VTN | 0.98 | 0.0213 | 1.01 | 0.9 |
| P11226 | MBL2 | 0.85 | 0.0107 | 0.87 | 0.02 |
| P02747 | C1QC | 0.85 | 0.0159 | 0.84 | 0.004 |
| P04003 | C4BPA | 0.82 | 0.0456 | / | / |
| P02746 | C1QB | 0.82 | 0.0165 | 0.86 | 0.008 |
| P07360 | C8γ | 0.80 | 0.0136 | 0.83 | 0.001 |
| P05155 | C1-INH | 0.78 | 0.0191 | 0.73 | 0.0001 |
| P13671 | C6 | 1.14 | 0.3212 | 1.13 | 0.03 |
| P02751 | FN | 1.70 | 0.0048 | 1.85 | 0.0004 |
| P02741 | CRP | 1.26 | 0.0199 | 1.37 | 0.0001 |


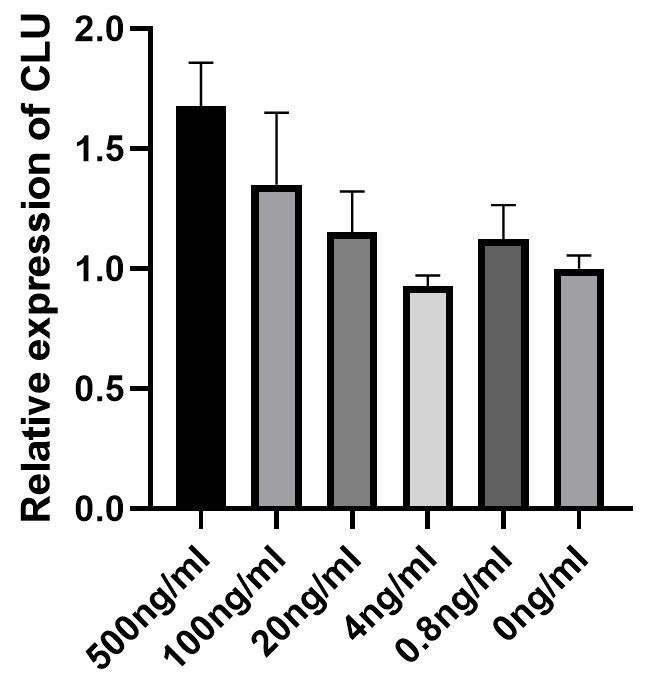


Fig. S2 Relative *mCLU* repression of TEV-1cells after treated with LPS for 24h. one-way ANOVA, n=3


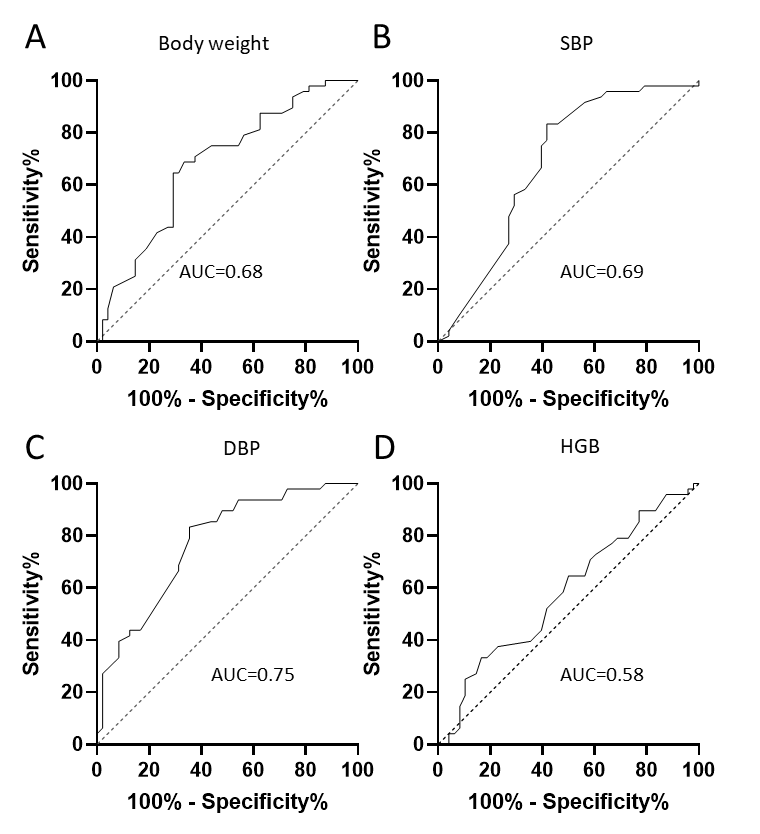


Fig. S3 ROC curve of four clinical factors for predicting HDP
